# Integrative Analysis of the Metabolome and Transcriptome of a Cultivated Pepper and Its Wild Progenitor Chiltepin (*Capsicum annuum* L. var. *glabriusculum*) Revealed the Loss of Pungency During *Capsicum* Domestication

**DOI:** 10.3389/fpls.2021.783496

**Published:** 2022-01-05

**Authors:** Bipei Zhang, Fang Hu, Xiaotao Cai, Jiaowen Cheng, Ying Zhang, Hui Lin, Kailin Hu, Zhiming Wu

**Affiliations:** ^1^College of Horticulture and Landscape Architecture, Zhongkai University of Agriculture and Engineering, Guangzhou, China; ^2^College of Horticulture, South China Agricultural University, Guangzhou, China

**Keywords:** pepper (*Capsicum annuum* L.), capsaicinoids, loss of pungency, metabolome and transcriptome, coexpression

## Abstract

Pungency is a unique characteristic of chili peppers (*Capsicum* spp.) caused by capsaicinoids. The evolutionary emergence of pungency is thought to be a derived trait within the genus *Capsicum*. However, it is not well-known how pungency has varied during *Capsicum* domestication and specialization. In this study, we applied a comparative metabolomics along with transcriptomics analysis to assess various changes between two peppers (a mildly pungent cultivated pepper BB3 and its hot progenitor chiltepin) at four stages of fruit development, focusing on pungency variation. A total of 558 metabolites were detected in two peppers. In comparison with chiltepin, capsaicinoid accumulation in BB3 was almost negligible at the early stage. Next, 412 DEGs associated with the capsaicinoid accumulation pathway were identified through coexpression analysis, of which 18 genes (14 TFs, 3 CBGs, and 1 UGT) were deemed key regulators due to their high coefficients. Based on these data, we speculated that downregulation of these hub genes during the early fruit developmental stage leads to a loss in pungency during Capsicum domestication (from chiltepin to BB3). Of note, a putative UDP-glycosyltransferase, GT86A1, is thought to affect the stabilization of capsaicinoids. Our results lay the foundation for further research on the genetic diversity of pungency traits during Capsicum domestication and specialization.

## Introduction

**FIGURE 1:**
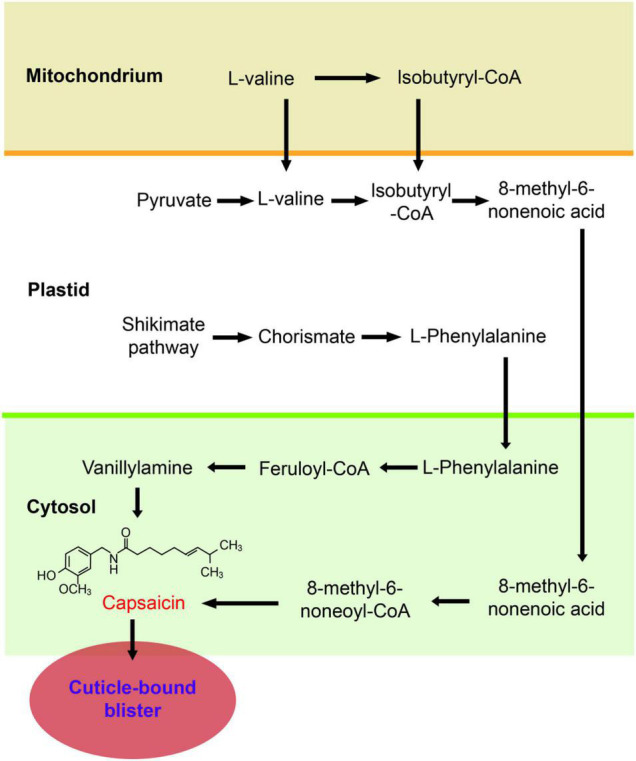
Capsaicinoid biosynthetic pathway (Naves et al., 2019). Schematic representations of the subcellular locations of the main steps involved in capsaicinoid biosynthesis. The convergence of two pathways is required- the phenylpropanoid pathway, which leads formation of vanillylamine, and the branched chain fatty acid pathway, which forms the 8-methyl-6-nonenoyl-CoA; the two final compounds are condensed by capsaicinoid synthase to form capsaicin. Abbreviations: ACS, acyl-CoA synthetase; AMT, aminotransferase; BCAT, branched-chain amino acid aminotransferase; C4H, cinnamate 4-hydroxylase; 4CL, 4-coumaroyl-CoA ligase; COMT, caffeoyl-CoA 3-O-methyltransferase; CS, capsaicinoid synthase; FatA, acyl-ACP thioesterase; HCHL, hydroxycinnamoyl-CoA hydratase lyase; PAL, phenylalanine ammonia-lyase.

Chili peppers (*Capsicum* spp.) are of great economic significance because these fruits are always served as vegetables and spices that are cultivated and consumed all over the world. The genus *Capsicum* contains more than 30 species, with five domesticated species (*Capsicum annuum* L., *Capsicum chinense* Jacq., *Capsicum baccatum* L., *Capsicum frutescens* L., and *Capsicum pubescens* Ruiz and Pavon) and several wild species (Qin et al., 2014; Pereira-Dias et al., 2019). The color, size, shape, aroma, pungency, etc., of the pepper fruits are important for quality classification (Kirschbaum-Titze et al., 2002). Unlike other members of the Solanaceae family, such as tomatoes (*Solanum lycopersicum* L.), eggplants (*Solanum melongena* L.), and potatoes (*Solanum tuberosum* L.), members of the genus *Capsicum* synthesize and accumulate uniquely pungent alkaloids known as capsaicinoids, mainly in the placentas of the fruits (Naves et al., 2019). It has been suggested that regular intake of capsaicinoids is beneficial for health due to their anti-inflammatory, antioxidant, antitumoral, and weight loss properties; therefore, chili peppers can be deemed functional foods (Conforti et al., 2007; Spiller et al., 2010; Chapa-Oliver and Mejía-Teniente, 2016; Varghese et al., 2017). Consequently, studies on the biosynthesis (Figure 1; Lang et al., 2009; Aza-González et al., 2011), metabolism (Kim et al., 2014; Naves et al., 2019), and gene regulation (Arce-Rodriguez and Ochoa-Alejo, 2017; Zhu et al., 2019) of capsaicinoids have become the focus of breeder and scholars worldwide.

In 1876, capsaicin, the most abundant capsaicinoid (and thus, the compound responsible for the characteristic spiciness of pepper fruits), was the first capsaicinoid identified; however, its chemical structure (C_18_H_27_NO_3_) and name (8-methyl-6-non-enoyl-CoA) were not fully established until 1919 (Stewart et al., 2005). To date, more than 20 capsaicinoids have been isolated from peppers, including capsaicin, dihydrocapsaicin, nordihydrocapsaicin, homodihydrocapsaicin, homocapsaicin, and others that have not been named (Mazourek et al., 2009). Based on bioinformatic analyses and genome sequencing, the capsaicinoid biosynthetic pathway has been largely elucidated (Mazourek et al., 2009; Kim et al., 2014). The first step is the phenylpropanoid pathway that is derived from phenylalanine and leads to vanillylamine, and the second is the branched chain fatty acid pathway that is derived from valine and leads to 8-methyl-6-non-enoyl-CoA. Some capsaicinoid biosynthetic genes (CBGs), such as *Pungent gene 1 (Pun1)* or *capsaicin synthase (CS)* (Stewart et al., 2005, 2007), putative aminotransferase (*p-AMP)* (Park et al., 2015), and ketoacyl-ACP reductase (*CaKR1*) (Koeda et al., 2019) are preferentially or specifically expressed in the placenta and regulated by the developmental stage of the fruit. The same as some transcription factors, especially *R2R3-MYB* gene (Wang et al., 2020), such as *pun3* or *CaMYB31* (Han et al., 2019; Zhu et al., 2019). Certain methods, such as gas chromatography-mass spectrometry (GC-MS), high-performance liquid chromatography (HPLC) or metabolome analysis (Liu et al., 2020), have been used to study metabolic diversity, the composition and contents of the major compounds in different cultivars and the different materials found in peppers. However, the correlation between phenotypes and metabolic profiles and genotypes of peppers are indeed far from revealing the evolution of pungency in *Capsicum*.

The evolutionary emergence of pungency is a derived trait within the *Capsicum* genus, since the capsaicinoid content greatly varies between *Capsicum* species (Carrizo García et al., 2016; M et al., 2016). To date, only a handful of studies have investigated how pungency varies during *Capsicum* domestication and specialization, much less the underlying mechanisms. Chiltepin (*C. annuum* var. *glabriusculum*) is an important genetic resource for both improving pepper crop cultivation due to its resistance to pathogens and higher genetic diversity than other cultivars (Rodelo-Urrego et al., 2015; Hayano-Kanashiro et al., 2016), and to better understand *Capsicum* evolution (Magdy et al., 2019; Pereira-Dias et al., 2019). Chiltepin is also an important source of capsaicinoids since it is considered to be the second hottest pepper in Mexico [100,000–200,000 Scoville heat units (SHUs)] after only the habanero chili pepper (*C. chinense*; 100,000–445,000 SHUs) (Hayano-Kanashiro et al., 2016). In 2014, the genomes of cultivated *C. annuum* cv. CM334 and cv. Zunla-1 and the wild progenitor chiltepin were made accessible (Kim et al., 2014; Qin et al., 2014). The genome size of chiltepin was estimated to be 3.07 Gb, which 34,476 genes were identified (Qin et al., 2014). This gives us the opportunity to further study the molecular mechanisms that regulate the biochemical pathways involved in pungency that vary during *Capsicum* domestication and specialization.

In this study, we performed comprehensive metabolomic and transcriptomic analyses between the cultivated pepper BB3 and its progenitor chiltepin using fruits collected during four different developmental stages. We aimed to identify the difference between the two peppers at the transcriptional and metabolite levels, elucidate the regulatory and metabolic pathways involved in capsaicinoid biosynthesis, and explore the loss of pungency during *Capsicum* domestication. These results will provide new insights into the flavor changes in pepper fruits for fruit quality improvement during pepper breeding.

## Materials and Methods

### Plant Materials and Sampling

The cultivated pepper BB3 (*C. annuum* L.) and its wild progenitor chiltepin (*C. annuum* var. *glabriusculum*) were used in this study. BB3 is a highly inbred line with normal fruits and slight pungence that is used as a maintainer line backcrossed with BA3 in the CMS system in our lab. Chiltepin is a semidomesticated or wild pepper that has been sequenced by our group and contains very small fruits that are considered the 2nd spiciest peppers in Mexico (100,000–200,000 SHUs) after the habanero chili pepper (*C. chinense*) (Hayano-Kanashiro et al., 2016); this pepper was kindly offered by Dr. Rafael F. Rivera-Bustamante. Plants were grown in the open field at the Zengcheng Experimental Station, South China Agricultural University (SCAU), Guangzhou, China (23° N, 113° E) in the spring of 2018. The pepper fruits were harvested at 4 different stages, including 11, 22, 33, and 55 DAP (Figure 1). All experiments contained three biological repeats, and each replicate was a pooled sample of 10 fruits of uniform size from 5 individual plants. In total, 24 samples were immediately frozen in liquid nitrogen and stored at –80°C for further metabolomic and transcriptomic analyses. The fruit widths, lengths, weights and flesh thicknesses were measured by using digital Vernier calipers and an electronic balance.

### Metabolome Analysis

Twenty-four fruit samples from BB3 and chiltepin with three independent biological replicates were used for metabolome analysis. The freeze-dried pepper fruits were crushed into power with a mixer mill (MM 400, Retsch) with a zirconia bead for 1.5 min at 30 Hz. Each powder sample was weighted 100 mg and extracted overnight at 4^°^C with 1.0 mL 70% aqueous methanol. Following centrifugation at 10, 000 g for 10 min, the supernatant extracts were absorbed using a CNWBOND Carbon-GCB SPE Cartridge (250 mg, 3 mL; ANPEL, Shanghai, China^1^) and filtrated through a SCAA-104 membrane (0.22 μm; ANPEL, Shanghai, China, see text footnote 1) and then a liquid chromatography-electrospray ionization-tandem mass spectrometry system was used for the relative quantification of widely targeted metabolites in dried pepper fruit samples. The treated extracts were analyzed using an LC-ESI-MS/MS system (UPLC, Shim-pack UFLC CBM30A, Shimadzu, Kyoto, Japan; MS, 6500 QTRAP, Applied Biosystems, Norwalk, United States) at Wuhan METWARE Biotechnology Co., Ltd. (Wuhan, China) following their standard procedures as fully described before (Chen et al., 2013; Zhu et al., 2018). A local database was generated by a proper combination of authentic standards and manual identification that was applied as one of the references.

The analytical conditions were as follows, HPLC: column, Waters ACQUITY UPLC HSS T3 C18 (1.8 μm, 2.1 mm × 100 mm); solvent system, water (0.04% acetic acid): acetonitrile (0.04% acetic acid); gradient program, 95:5 V/V at 0 min, 5:95 V/V at 11.0 min, 5:95 V/V at 12.0 min, 95:5 V/V at 12.1 min, 95:5 V/V at 15.0 min; flow rate, 0.40 mL/min; temperature, 40^°^C; injection volume: 2 μL. The MS was operated in positive and negative ion modes. The ESI source operation parameters were as follows: ion source, turbo spray; source temperature 500^°^C; ion spray voltage (IS) 5,500 V; ion source gas I (GSI), gas II(GSII), curtain gas (CUR) was set at 55, 60, and 25.0 psi, respectively; the collision gas (CAD) was high. Based on the MWDB (metware database) and metabolite information public database, the metabolite is annotated only if five indexes (Q1, Q3, Retention time, Declustering potential and Collision energy) are 100% identical to both databases. Metabolite quantification was performed using a multiple reaction monitoring method (MRM). The MRM transition information is provided in Supplementary Table 2. Data were processed using Analyst 1.6.3 software. The screening of significant DAMs was carried out by combining the VIP value of the OPLS-DA model and fold change between the two samples. The screening criteria were as follows: (1) fold change ≥ 2 or fold change ≤ 0.5 and (2) metabolites with VIP ≥ 1 (Yuan et al., 2018).

### Transcriptome Analysis

Total RNA was extracted from frozen tissues with TRIzol reagent (Invitrogen, Carlsbad, CA, United States) following the manufacturer’s instructions and then subjected to quantity and quality assessment by agarose gel electrophoresis, an Agilent 2100 Bioanalyzer (Agilent Technologies, Palo Alto, CA, United States) and a NanoPhotometer UV-Vis Spectrophotometer (Implen GmbH, Münchenm, Germany). mRNA was purified by magnetic beads with oligo (dT), and then first- and second-strand complementary DNA was synthesized. The double-stranded cDNA was purified, and sequencing adapters were added followed by cleavage into short fragments. The resulting cDNA libraries were sequenced on an Illumina HiSeq 2500 system by Wuhan METWARE Biotechnology Co., Ltd. (Wuhan, China). Clean reads obtained from the raw reads were processed to remove those containing adapters and low-quality reads. The clean reads were then mapped to the pepper reference genome (Zunla1 version 2.0) using HISAT2 (Kim et al., 2015; Liu et al., 2020). In this project, fragments per kilobase of transcript per million mapped reads (FPKM) was used as an index to measure the level of transcript or gene expression. The screening criteria for the DEGs between each sample were | log_2_FC| ≥ 1 and FDR < 0.05 (Love et al., 2014). Three replicates were performed for each sample. GO enrichment (Ashburner et al., 2000) and KEGG pathway (Kanehisa and Goto, 2000) enrichment analyses of the DEGs were performed using the OmicShare tools, a free online platform for data analysis.^2^

### Transcriptome and Metabolism Conjoint Analysis

For coexpression network analysis, the WGCNA package was used (Langfelder and Horvath, 2008; DiLeo et al., 2011). The DEGs and DAMs in the same group were mapped together to the KEGG pathway map and screened using a *P*-value < 0.05 to identify significant relationships. A total of 7,946 unigenes with FPKM values > 1 were used to perform WGCNA. All FPKM values were transformed into the topological overlap matrix (TOM), and each gene was analyzed by hierarchical clustering. Different genes were categorized by the dynamic tree cut method into distinct coexpression modules, and the minimum number of genes in each coexpression module was set to 50. With 0.7 as the boundary, the coexpression modules with similar clustering were merged, and the correlation between different modules and the degree of association (module membership, MM) of the genes within the modules were calculated. The key modules related to the alkaloid content in the fruits were used for subsequent analysis. Finally, Cytoscape_V.3.7.1 was used to draw the visualized regulatory network map.

### Statistical Analysis

The gene expression and metabolite data were standardized to the Z score by using TBtools software (Chen et al., 2020). We analyzed and compared the differences among four sample groups (BB3-11DAP_vs_Chiltepin-11DAP, BB3-22DAP_vs_Chiltepin-22DAP, BB3-33DAP_vs_Chiltepin-33DAP, BB3-55DAP_vs_Chiltepin-55DAP). The physiological data are expressed as the positive and negative standard deviations of the average values of the three repeated samples. SPSS software (v18.0) was used for analysis of variance.

### Relative Gene Expression Verification by Quantitative Real-Time Polymerase Chain Reaction

Expression pattern analysis was investigated using quantitative real-time polymerase chain reaction (qRT-PCR). qRT-PCR amplification was carried out using SYBR Green Pro Taq HS (Takara, Dalian, China) according to the manufacturer’s instructions. qRT-PCR was performed on a CFX96 real-time PCR system (Bio-Rad, Alfred Nobel Drive Hercules, CA, United States) using the following program: 95°C for 30 s, followed by 40 cycles of 95°C for 5 s and 60°C for 30 s. The *CaActin* (AY496112) pepper gene was amplified as a control gene. Three biological replicates and three measurements for each replicate were performed under identical conditions. Analysis of the relative mRNA expression data was performed using 2^–ΔΔCt^ (Livak and Schmittgen, 2001). All primers used in this study are listed in Supplementary Table 1.

## Results

### Phenotypic Comparison Between the Wild Progenitor Chiltepin and the Cultivated Pepper BB3

**FIGURE 2:**
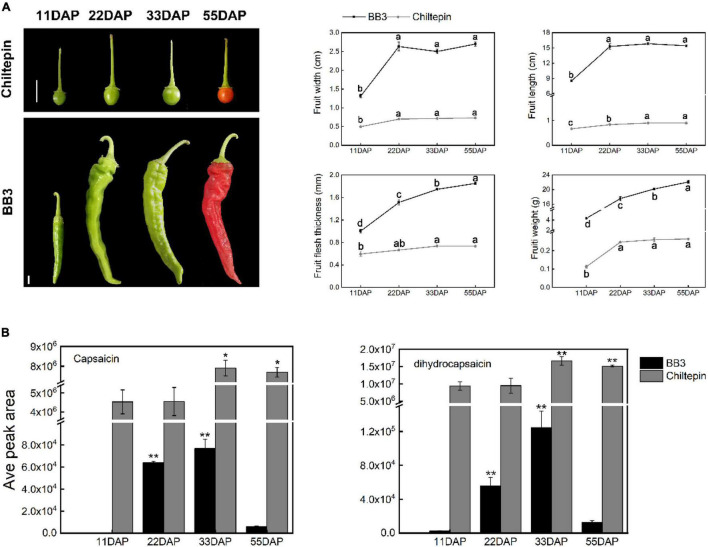
Comparison of the phenotypes and capsaicin and dihydrocapsaicin contents between *C. annuum* cv. BB3 and the progenitor chiltepin at different sampling stages. **(A)** Changes in fruit appearance of BB3 and chiltepin at 11, 22, 33, and 55 DAP. Scale bars indicate 1 cm. Values represent the means ± SEs of three replicates. Columns labeled with different letters indicate statistically significant differences (*P* ≤ 0.05, one-way ANOVA with *post hoc* comparisons). **(B)** The contents of capsaicin and dihydrocapsaicin in the fruits of BB3 and chiltepin at 11, 22, 33, and 55 DAP. Data are represented as the means ± SD from three technical replicates (*P ≤ 0.05, **P ≤ 0.01; Student’s *t*-test).

To discuss pepper domestication and specialization (especially with respect to pungent diversification), it is important to provide a brief morphological comparison of the cultivated pepper BB3 and its progenitor chiltepin. Distinct differences in size phenotype, including width, length, weight and fruit flesh thickness, were observed (Figure 2A). A phylogenetic tree further revealed that the wild and cultivated peppers are genetically distinguishable (Qin et al., 2014). The chiltepin fruit is small with round dark green berries in their immature state that turn red upon maturation and are 6–8 mm in diameter. In contrast, BB3 is 13 (11 days after pollination, 11 DAP) –18 (33 DAP) times larger in length and 38 (11 DAP) –83 (55 DAP) times larger in weight than its progenitor.

Capsaicinoids are responsible for the pungent sensation of Capsicum fruits (Tewksbury et al., 2008). Interestingly, the capsaicinoid content reached its peak at 33 DAP in both chiltepin and BB3 fruits rather than at the red mature fruit stage (55 DAP), as was also found in previous reports (Conforti et al., 2007; Figure 2B). Notably, BB3 showed a substantial decline in capsaicinoid contents compared with chiltepin, suggesting that a reduction in capsaicinoid biosynthesis occurred during Capsicum fruit development (Figure 2B).

### Characterization of the Metabolites in the Two Peppers During Fruit Development

**FIGURE 3:**
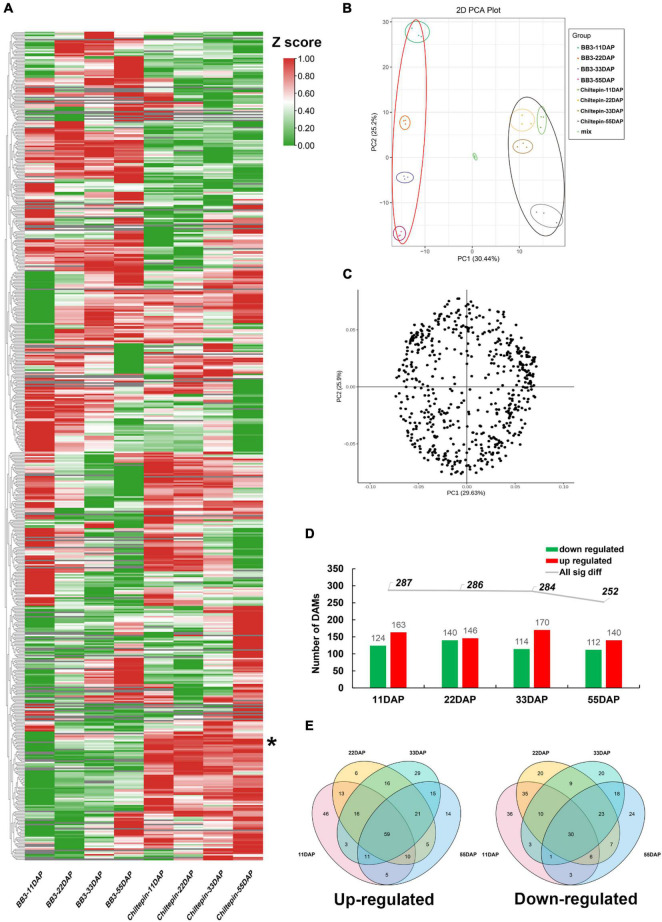
Metabolome data analysis in *C. annuum* cv. BB3 and the progenitor chiltepin at different sampling stages. **(A)** Clustering heat map of metabolites. Each differently colored rectangle on the left side of the heatmap represents one metabolite with its specific information shown in Supplementary Table 2. The values in the image are the averages of three biological duplicate samples. The Z-score transformed response indicates the accumulation ratio of each metabolite, from low (green) to high (red). * indicated capsaicinoids. **(B)** PCA of the metabolites identified from the fruits of BB3 chiltepin at 11, 22, 33, and 55 DAP. Score plots were derived using LC-ESI-MS/MS datasets. The X axis represents PC1, and the Y axis represents PC2. Each sample has three biological duplicates and is represented on the plot by a unique symbol. **(C)** Loading pot of the metabolites identified from the fruits of BB3 chiltepin at 11, 22, 33, and 55 DAP. Score plots were derived using LC-ESI-MS/MS datasets. The X axis represents PC1, and the Y axis represents PC2. Each sample has three biological duplicates and is represented on the plot by a unique symbol. **(D)** Total number of DAMs from pairwise comparisons of BB3 and chiltepin fruits (BB3_vs_Chiltepin) at different developmental stages. **(E)** Venn diagrams of significantly upregulated (left) and downregulated (right) metabolites in the fruits of BB3_vs_Chiltepin at 11, 22, 33, and 55 DAP.

The conspicuous differences between the fruit morphology and pungency of these two peppers prompted us to further explore the correlation between fruit phenotype and metabolic profile. To this end, we attempted widely targeted liquid chromatography-electrospray ionization tandem mass spectrometry (LC-ESI-MS/MS)-based metabolite profiling of chiltepin and BB3 spanning fruit development and maturation (see section “Materials and Methods”). In total, 558 distinct annotated metabolites were identified and classified into 11 main groups, which included a total of 25 metabolites detected in only BB3 fruits, while 12 metabolites existed specifically in chiltepin (Figure 3A and Supplementary Table 2). As expected, the accumulation of two detected capsaicinoids (capsaicin and dihydrocapsaicin) were much higher in chiltepin than in BB3 throughout fruit maturation, implying an loss of pungency during Capsicum domestication. Strikingly, we noticed that several hexoside had a similar accumulation profile to that of the capsaicinoids in the two peppers, suggesting their functional relevance in Capsicum pungency domestication. Principal component analysis (PCA) of the metabolite data (using the normalized responses that accounted for 55.64% of the variation with a significance level of 0.01) clearly separated the two peppers with respect to their metabolite compositions and contents (Figures 3B,C). This result showed that differences existed between these two peppers at the metabolite level.

To confirm metabolite gain and loss during the domestication and specialization of *Capsicum*, several statistical tests were performed. We selected metabolites with a fold change ≥2 (up) or ≤0.5 (down) and a variable importance in projection (VIP) value > 1 in BB3 fruits from the OPLS-DA model compared to those in chiltepin, which included four groups. The differential accumulation of metabolites (DAMs) in the two peppers during fruit development is displayed in Figures 3D,E and Supplementary Table 3. The results showed that up to 287 (124 down and 163 up), 286 (140 down and 146 up), 284 (114 down and 170 up) and 252 (112 down and 140 up) metabolites exhibited differential contents in BB3_vs_Chiltepin at the different fruit developmental stages from 11 to 55 DAP (Figure 3D). Through DAM Venn analysis, 59 upregulated and 30 downregulated DAMs were discovered among the two cultivars throughout fruit development (Figure 3E). Metabolites such as lipids, nucleotides and their derivatives and phenolamides preferentially accumulated in the immature fruits of BB3_vs_Chiltepin, whereas mature tissues had higher levels of amino acids and their derivatives, flavonoids, organic acids, polyphenols, and vitamins. These specific metabolites likely reflect the temporal differentiation of pepper metabolism during pepper fruit development.

### Transcriptional and Functional Enrichment Analyses of Chiltepin and BB3

**FIGURE 4:**
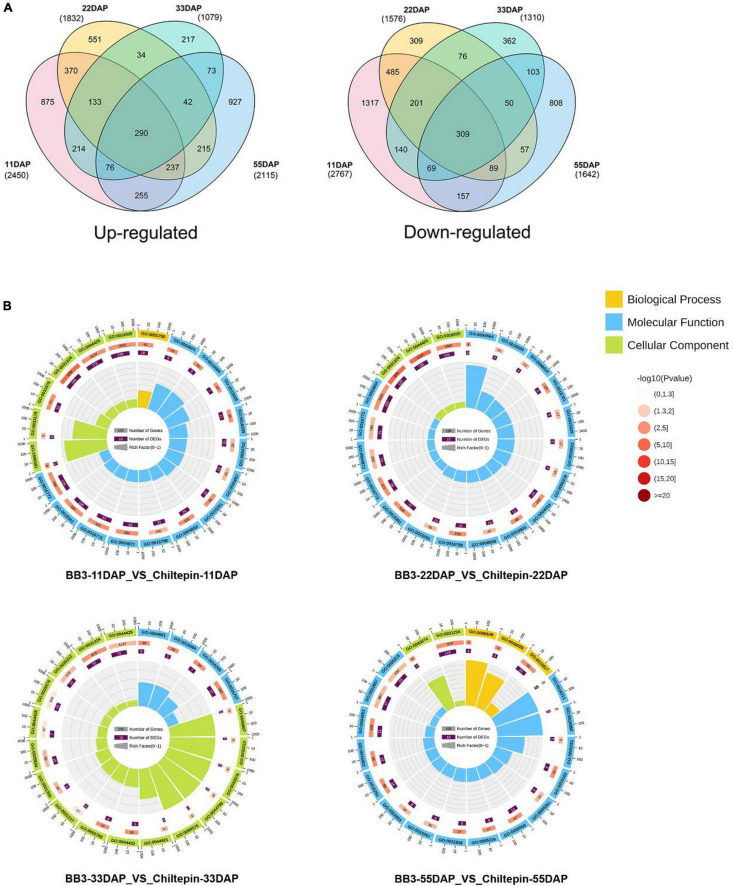
Transcriptome data analysis in *C. annuum* cv. BB3 and the progenitor chiltepin at different sampling stages. **(A)** Venn diagram indicating the number of DEGs in BB3_vs_Chiltepin at different sampling stages. **(B)** The top 20 pathways with the most significant Q value. First circle: the top 20 GO terms of the enrichment; outside the circle is the coordinate ruler of the number of genes. Different colors represent different ontologies. Second circle: the number of GO terms in the background gene and the Q value. The more genes there were, the longer the bar, the smaller the Q value, and the redder the color. Third circle: dark purple represents the differential gene, and the box shows the specific value Fourth circle: the rich factor value of each GO term (the number of differential genes in the GO term divided by the total number of genes) and the background grid lines; each grid represents 0.1.

The mRNA expression profiles of chiltepin and BB3 at the four fruit developmental stages were compared using the Illumina paired-end sequencing method with three biological replicates per time point. The resulting sets yielded more than 1.13 × 10^9^ clean reads, with over 80% mapped to the *Capsicum* genome (Supplementary Table 4). First, the differentially expressed genes (DEGs) were identified with the criteria of a false discovery rate (FDR) < 0.01 and a | log_2_FC (fold change) | > 1. The total number of DEGs between the two peppers was 5,217 during the early developmental stage (11 DAP) but decreased to 2,389 at 33 DAP, indicating that the changes in the gene expression levels occurred very early during development (Figure 4A and Supplementary Table 5).

**FIGURE 5:**
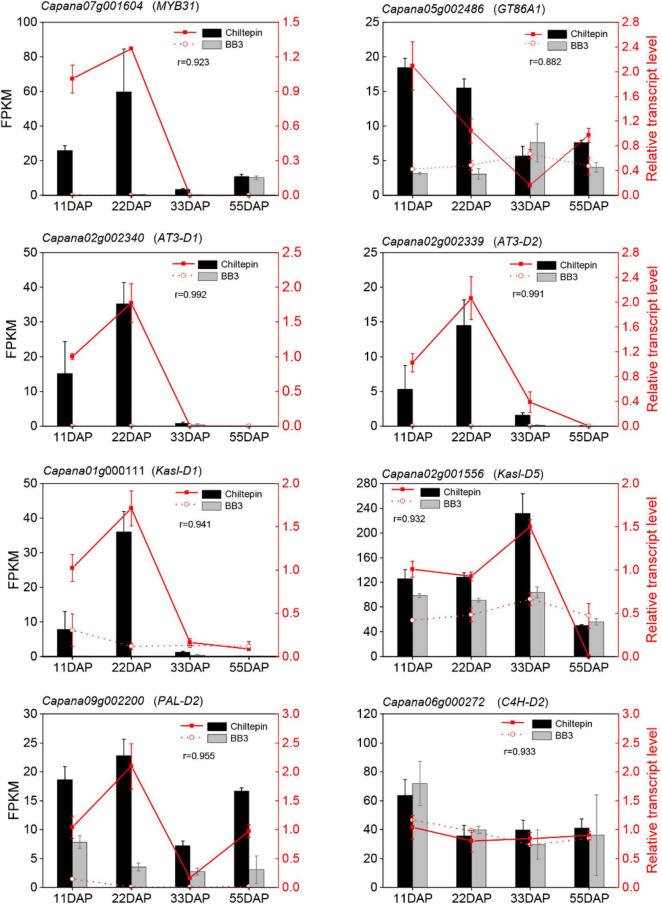
Quantitative real-time polymerase chain reaction (qRT-PCR) verify capsinoids biosynthesis-relative genes. Correlation analysis showed a good correlation between the RNA-seq data and the qRT-PCR data. The left y-axis indicates the corresponding expression data of RNA-seq (cylindrical). The right y-axis shows the relative gene expression levels detected by qRT-PCR (red lines). The x-axis shows the different stages of maturity. Pearson’s product moment correlation coefficient r.

Next, we annotated the Gene Ontology (GO) functional enrichment analyses of up- and downregulated transcripts comparing chiltepin with BB3 at each stage to assess their underlying biological significance during *Capsicum* domestication and specialization. The DEGs were divided into three main categories, namely, molecular function, biological process, and cellular component, and there were 49 GO classification subcategories. Herein, 859, 648, and 213 unigenes were assigned to the biological process, molecular function, and cellular component terms, respectively. These genes were further classified into 48 functional subcategories based on mapped homology. Genes in the biological process category were primarily matched and classified into metabolic processes, cellular processes, and single-organism processes. In the molecular function term, most of the unigenes exhibited catalytic activity and catalytic, transferase and transporter activity (Figure 4B and Supplementary Figure 1). qRT-PCR experiments validated the RNA-seq results using eight randomly selected differentially expressed CBGs. The expression patterns of these genes according to qRT-PCR were consistent with those determined by RNA-seq (Figure 5).

### Integrated Analysis of Capsaicinoids Accumulation During *Capsicum* Domestication

**FIGURE 6:**
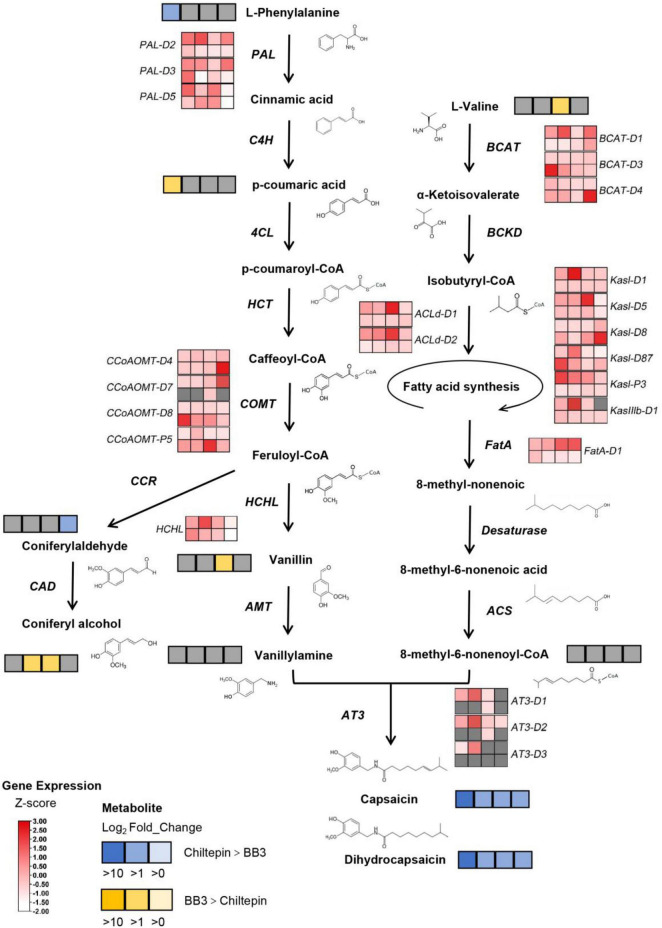
Comparison of the differential metabolites and differential genes in the BB3 and chiltepin capsaicinoid synthesis pathways. Metabolites: Significant metabolite changes (VIP > 1) observed from BB3_vs_Chiltepin are shown in the metabolic map. Metabolites shown as squares are DAMs detected between BB3 and chiltepin. Blue squares indicate metabolites that had lower contents in BB3 than in chiltepin, and the darker the color, the larger the difference. Similarly, the yellow squares indicate metabolites that had higher contents in BB3 than in chiltepin, and the darker the color, the larger the difference. The gray squares indicate that the metabolite showed no difference between the two peppers. From left to right: 11, 22, 33, and 55 DAP. Genes: Significant DEGs observed in BB3 (lower lane) vs. chiltepin (upper lane) were determined at | log_2_FC| ≥ 1 and FDR < 0.05. Z-score transformed FPKM indicates the relative expression level of each gene, from low (white) to high (red). Gray squares indicate that the expression levels of those genes were undetectable. From left to right: 11, 22, 33, and 55 DAP.

The metabolic components were mapped onto the capsaicinoid metabolism pathway by combining the metabolic components and related genes in the Kyoto Encyclopedia of Genes and Genomes (KEGG) pathway. The differentially annotated metabolites, together with the differentially annotated genes, are indicated on the integrated metabolic map (Figure 6 and Supplementary Table 6). The acyltransferase AT3 is thought to be a key enzyme involved in capsaicinoid biosynthesis. Notably, *AT3* showed extremely low expression levels in BB3 throughout the fruit developmental stages, which was thought to lead to the final loss of capsaicinoids, despite a weak recovery at 33 DAP.

**FIGURE 7:**
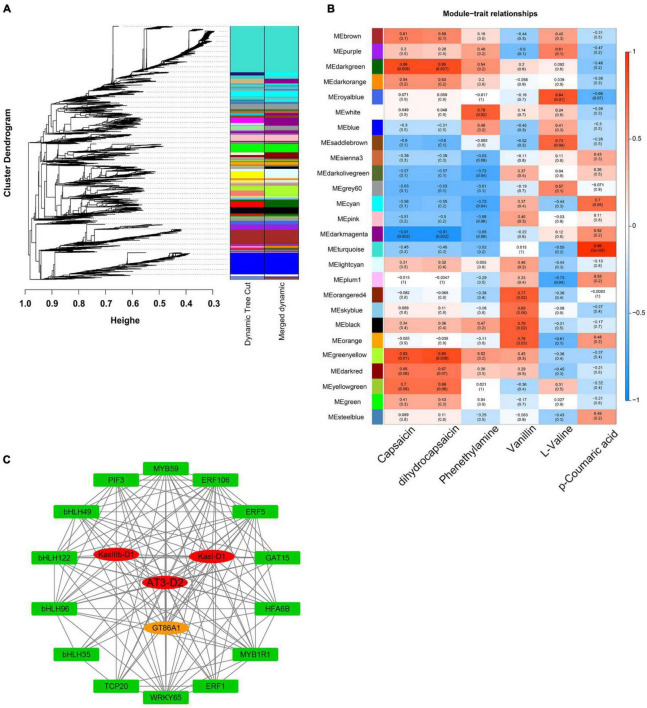
WGCNA of pepper fruits during ripening. **(A)** Hierarchical cluster tree showing coexpression modules identified by WGCNA. Each leaf in the tree represents one gene. The major tree branches constitute 26 modules labeled by different colors. **(B)** Module-alkaloid correlations, where each row corresponds to a module. The left panel shows the 26 modules, and the right panel shows a color scale for module/trait correlations ranging from -1 to 1. **(C)** Coexpression networks of TFs and structural genes involved in capsaicinoid metabolism. The network includes TFs and structural genes from the greenyellow module.

To further reveal the gene regulatory network of capsaicinoid synthesis in pepper fruits during *Capsicum* domestication, we performed a weighted gene coexpression network analysis (WGCNA) using 7,946 non-redundant DEGs (with DAFs PCC ≥ 0.90 or ≤ –0.90) (Supplementary Table 7). These DEGs were clustered into 26 major tree branches, each of which represents a module (each labeled with a different color) (Figure 7A and Supplementary Table 8).

Correlations were investigated among the expression patterns of each module and capsaicinoid biosynthesis pathway. The results showed that module “greenyellow” had a high correlation with the final production of capsaicinoids, indicating that this module was closely related to the loss of pungency during *Capsicum* domestication. The “greenyellow” module contained a total of 412 genes (Supplementary Table 9), including the genes *AT3* (*Capana02g002339*), *Kasl* (*Capana01g000111*), and *KasIII* (*Capana01g004020*). Further, a gene correlation network of 18 genes from the “greenyellow” module was constructed and were regarded as hub genes according to their degree of connectivity (Figures 7B,C).

Among these 18 hub genes (Supplementary Table 10), we found one UDP-glycosyltransferase and 14 TFs, which include the MYB, bHLH, AP2/ERF, TCP, PIF families and the WRKY protein. These TFs, as highly connected hub genes, may have regulatory effects on the capsaicinoid biosynthesis pathway in pepper fruits during *Capsicum* domestication.

## Discussion

In the present study, we applied a metabolomics approach along with transcriptomics analysis to assess the various changes in two peppers (the cultivated pepper BB3 and its progenitor chiltepin) at four different fruit development stages, focusing on pungency variation.

### Metabolites and Gene Expression Temporally Differ Between the Two Peppers During the Early Stage of Fruit Development

The contents of capsaicinoids, which contribute to pepper fruit pungency, are highly dynamic during fruit development (Barbero et al., 2014; Fayos et al., 2017). Capsaicinoids begin to accumulate from the early stages (11 DAP) of fruit development, peak at approximately 33 DAP, and then decrease sharply in cultivated BB3 while stabilizing at high levels in the progenitor chiltepin (Figure 1B).

The changes in metabolites and gene expression were distinct between the two peppers at each developmental stage (11, 22, 33, and 55 DAP). The heat map (Figure 3A and Supplementary Table 2), gene expression levels (Figures 4, 5 and Supplementary Table 5), and biosynthetic pathways (Figure 6 and Supplementary Table 6) suggested that transferase and kinase activities mainly determined capsaicinoid variation between the two peppers at the early stage (approximately 11 DAP), followed by increased catalytic and glycosyltransferase activities to accumulate and then stabilize the capsaicinoid contents in chiltepin at the mid-stage (22–33 DAP). Moreover, 412 DEGs associated with capsaicinoid biosynthesis were selected for further coexpression analysis as variables between the two peppers at each stage, including glycosyltransferases and acyltransferases (Figure 7 and Supplementary Table 9).

### The Downregulation of Multiple Structural Genes in the Capsaicinoid Biosynthesis Pathway Leads to a Loss in Pungency in Cultivated BB3

In cultivated BB3, a loss of pungency can occur through a variety of different mutations that affect gene expression in the biosynthetic pathway, of which a well-known *Pun1* locus where *AT3* has been isolated has a qualitative effect on pungency accumulation (Stewart et al., 2005; Barbero et al., 2014). *AT3* acylates vanillylamine with a fatty acid to form a capsaicinoid and is the most downstream gene in the capsaicinoid biosynthesis pathway (Stewart et al., 2005; Aza-González et al., 2011; Tanaka et al., 2017). In our study, the expression of *AT3* was negligible in BB3 at all developmental stages, while it was strongly upregulated from 11 to 55 DAP in chiltepin (Figure 6 and Supplementary Table 6), which is consistent with previous findings in hot peppers (Stewart et al., 2007).

Compared to chiltepin, in BB3, CBGs have lower expression levels (PAL, BCAT, ACLd, Kas, FatA, and HCHL); moreover, some have lower expression levels with temporally restricted expression (COMT) in addition to *AT3*; and one gene encoding a putative UDP-glycosyltransferase, *GT86A1*, was significantly downregulated at each stage. Thus, we proposed that this glycosyltransferase enzyme (UGT) functions to stabilize capsaicinoids *via* moiety modification apart from its suggested activities in the metabolic detoxification of capsaicin (Ahn et al., 2011).

### The Coexpression Pattern of Capsaicinoid Biosynthesis-Related TFs Underlies the Loss of Pungency During *Capsicum* Domestication

Thus, pungency appears to be under transcriptional control, as shown by the expression levels of capsaicinoid biosynthesis-related TFs at different developmental stages (Minwoo et al., 2001; Tanaka et al., 2017; Naves et al., 2019). Based on a WGCNA of the DEGs involved in the capsaicinoid biosynthesis pathway, we narrowed down 18 hub genes into a highly correlated key module, in which 14 TFs (4 bHLHs, 3 ERFs, 2 MYBs, 1 WRKY, 1 PIF, 1 TCP, 1 GAT, and 1 HFA) were considered to be involved in the upstream regulation of four structural genes (Figure 7C).

**FIGURE 8:**
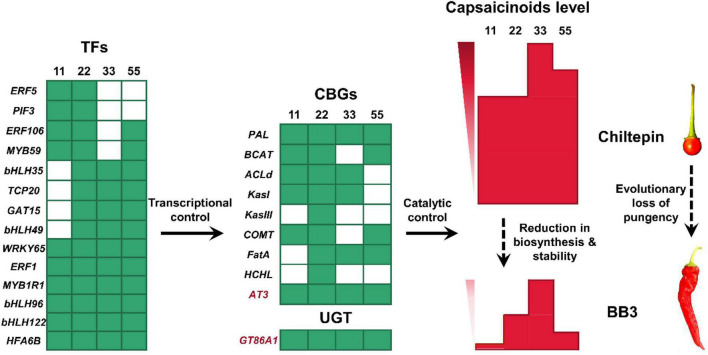
Schematic representation of the loss of pungency from chiltepin to BB3. As positive upstream regulators, 14 TFs transcriptionally control the expression level of 10 enzyme genes (9 CBGs and 1 UGT) in capsaicinoid accumulation pathways, of which *AT3* (responsible for synthesis) and *GT86A1* (responsible for stability) are strongly correlated with capsaicinoid levels in pepper fruits. From chiltepin to BB3, 14 TFs were significantly downregulated at the very early fruit developmental stage (green cells), followed by the low expression levels of 10 downstream structural enzyme genes (green cells). As a result, the final capsaicinoid content in BB3 dramatically decreased due to its reduced synthesis ability and stability (red columns). The numbers 11, 22, 33, and 55 correspond to 11 DAP, 22 DAP, 33 DAP and 55 DAP, respectively.

The levels of vanillylamine and 8-methyl-6-non-enoyl-CoA, two direct precursors for AT3 in the last step of capsaicinoid biosynthesis, were comparable in BB3 and chiltepin (Figure 6). This result suggests that the final downregulation of AT3 (both its concentration and activity) during capsaicinoid biosynthesis accompanied by the subsequent reduced capsaicinoid stabilization ultimately determines the loss of pungency in BB3. It is therefore conceivable that certain TFs and capsaicinoid stabilizers are involved in this coevolution event (Figure 8).

## Conclusion

We linked significant changes in pungency-related metabolites with the expression of selected enzyme genes and associated TFs at four stages of pepper fruit development with respect to the loss of pungency from chiltepin to BB3. Our data revealed that the roles of 18 key coexpression hub genes and functional biological pathways were associated with the downregulation of capsaicinoid accumulation in BB3_vs_Chiltepin. Significantly, 14 TFs that control the expression levels of AT3 and GT86A1 ultimately determined the loss of pungency in BB3. Thus, this study provides a foundation for further research to investigate the transcriptional regulatory networks of capsaicinoid biosynthesis in peppers and offers mechanistic insights into the domestication and specialization of plant secondary metabolism. However, the exact molecular mechanisms that connect the hub genes and the evolutionary route need further exploration.

## Data Availability Statement

High-throughput sequencing data generated in this study are publicly available. This data can be found on NCBI Sequence Read Archive (SRA) under the project accession number PRJNA779212.

## Author Contributions

BZ, FH, and XC: investigation, visualization, writing-original and draft, and writing-review and editing. JC, XC, and YZ: data curation, writing-review and editing, and formal analysis. KH and ZW: funding acquisition, project administration, and writing-review and editing. BZ and ZW: conceptualization, supervision, writing-original and draft, and writing-review and editing. All authors read, corrected, and approved the manuscript.

## Conflict of Interest

The authors declare that the research was conducted in the absence of any commercial or financial relationships that could be construed as a potential conflict of interest.

## Publisher’s Note

All claims expressed in this article are solely those of the authors and do not necessarily represent those of their affiliated organizations, or those of the publisher, the editors and the reviewers. Any product that may be evaluated in this article, or claim that may be made by its manufacturer, is not guaranteed or endorsed by the publisher.

## Footnotes

1
https://www.anpel.com.cn

2
https://www.omicshare.com/tools

## Supplementary Material

Supplementary Figure 1 GO enrichment bar graph. The first 20 GO terms with the smallest Q values are plotted. The ordinate is the GO term, and the abscissa is the percentage of the number of GO terms compared to the total number of differential genes. The darker the color, the smaller the Q value. The value is the GO term quantity and Q value.

Supplementary Figure 2 Secondary mass spectrum of capsaicin and dihydrocapsaicin standard products. Panel **(A)** is capsaicin and panel **(B)** is dihydrocapsaicin.

## Abbreviations

ACLd: acyl carrier protein
BCAT: branched-chain amino acid aminotransferase
*CaKR1*: ketoacyl-ACP reductase
CBGs: capsaicinoid-biosynthetic genes
COMT: caffeoyl-CoA 3-O-methyltransferase
*CS*: capsaicin synthase
DAMs: differential accumulation of metabolites
DAP: days after pollination
DEGs: differentially expressed genes
FatA: acyl-ACP thioesterase
FDR: false discovery rate
FPKM: fragments per kilobase of transcript per million mapped reads
GC-MS: gas chromatography-mass spectrometry
GO: gene ontology
HCHL: hydroxycinnamoyl-CoA hydratase lyase
HPLC: high performance liquid chromatography
KEGG: kyoto encyclopedia of genes and genomes
OPLS-DA: orthogonal partial least-squares discrimination analysis
PAL: phenylalanine ammonia-lyase
*p-AMP*: putative aminotransferase
PCA: principal component analysis
*Pun1*: pungent gene 1
qPCR: real-time polymerase chain reaction
SHU: Scoville heat units
TOM: topological overlap matrix
UGT: UDP-glycosyltransferases
VIP: variable importance in projection
WGCNA: weighted gene co-expression network analysis.
