# Supplementary figures and images for: Integrative Analysis of the Metabolome and Transcriptome of a Cultivated Pepper and Its Wild Progenitor Chiltepin (Capsicum annuum L. var. glabriusculum) Revealed the Loss of Pungency During Capsicum Domestication
Source: Front Plant Sci. 2022 Jan 5;12:783496. doi: 10.3389/fpls.2021.783496 (PMC8767146; doi:10.3389/fpls.2021.783496)

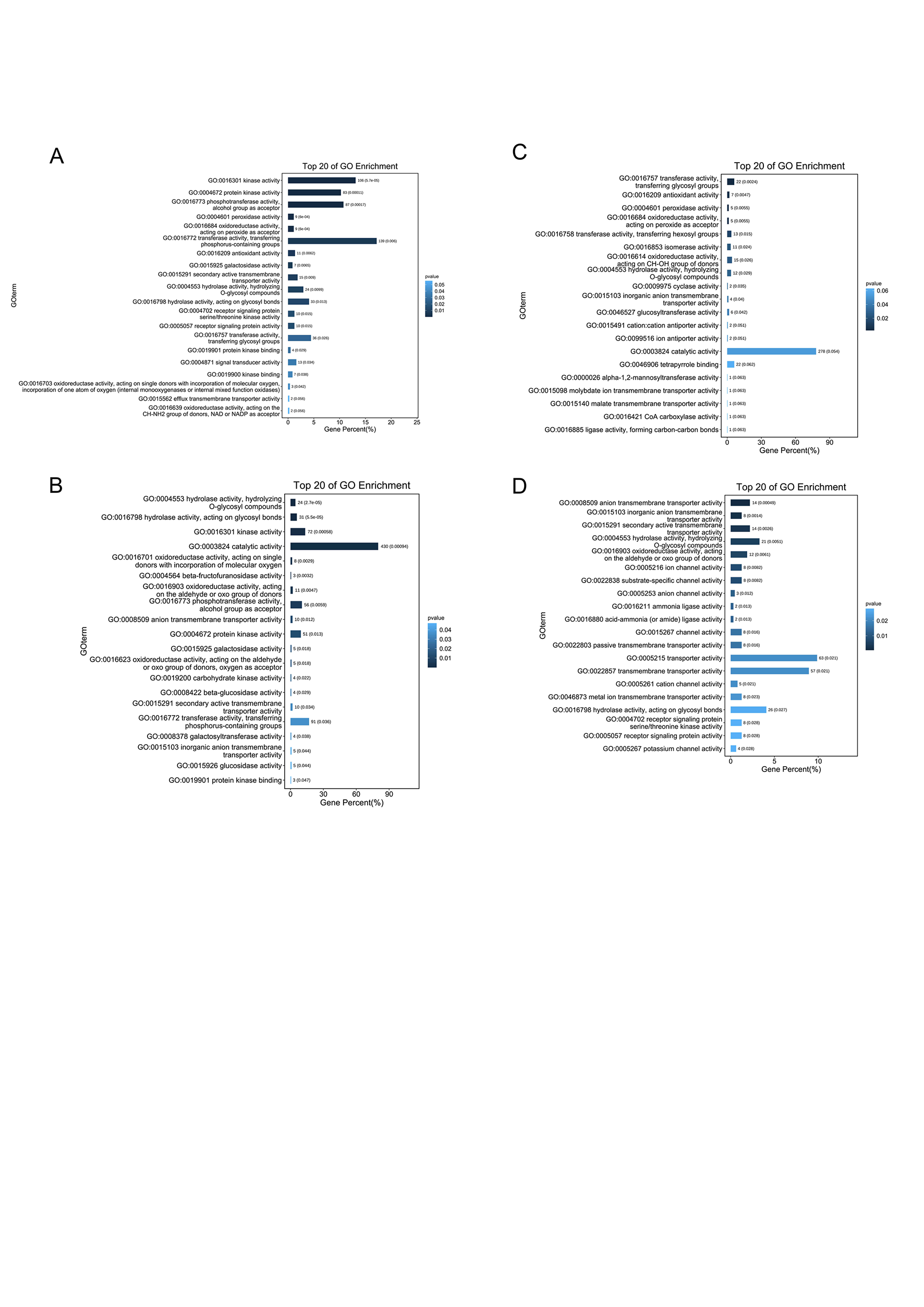

Supplement: Supplementary Figure 1 — GO enrichment bar graph. The first 20 GO terms with the smallest Q values are plotted. The ordinate is the GO term, and the abscissa is the percentage of the number of GO terms compared to the total number of differential genes. The darker the color, the smaller the Q value. The value is the GO term quantity and Q value. [file Image_1.TIF]

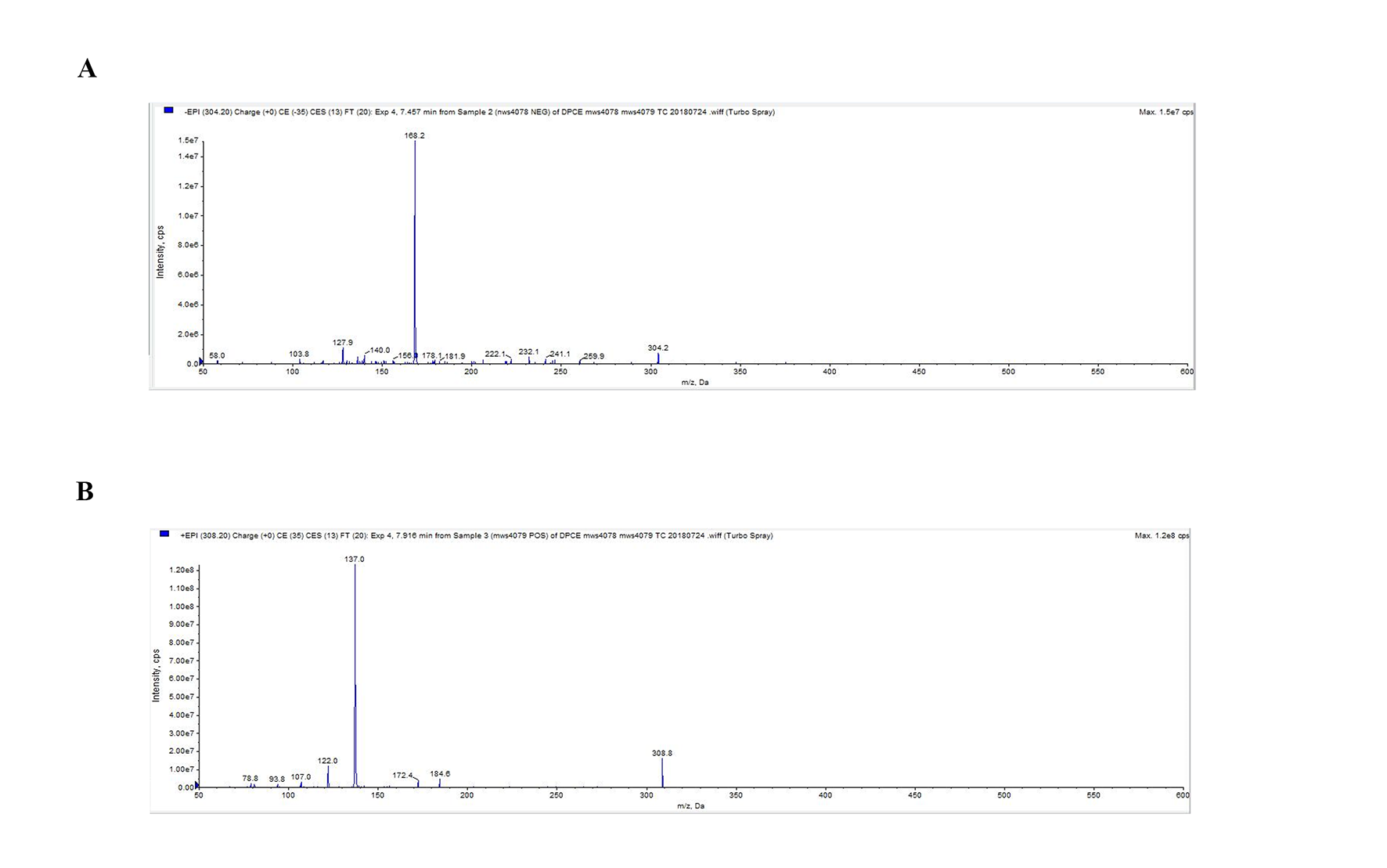

Supplement: Supplementary Figure 2 — Secondary mass spectrum of capsaicin and dihydrocapsaicin standard products. Panel (A) is capsaicin and panel (B) is dihydrocapsaicin. [file Image_2.TIF]
